# Towards a sustainable HIV response: strengthening Zimbabwe's domestic financing for HIV programs amid declining donor support

**DOI:** 10.3389/frhs.2025.1558992

**Published:** 2025-05-30

**Authors:** Godfrey Musuka, Tatenda Makoni, Tafadzwa Dzinamarira

**Affiliations:** ^1^ International Initiative for Impact Evaluation, Inc., Harare, Zimbabwe; ^2^ Zimbabwe National Network of People Living with HIV, Harare, Zimbabwe; ^3^ ICAP, Harare, Zimbabwe

**Keywords:** HIV response, domestic resource mobilization, sustainable health systems, health financing, Zimbabwe

Zimbabwe, a country with a population of approximately 16 million, has experienced substantial economic challenges over the past few decades. However, recent projections suggest a more optimistic outlook, with expectations that the economy will continue to grow for the next five years, and the country is on track to attain middle-income status by 2030. Notably, according to the World Bank Group, Zimbabwe has become one of the fastest-growing economies in Southern Africa, with growth rates of 6.1% in 2022 and 5.3% in 2023, driven by a robust expansion in agriculture, mining, and services fueled by remittances from its diaspora and a robust mining sector of key minerals such as lithium, platinum, gold and other base metals (1). This shift in economic status is poised to provide new opportunities, particularly for strengthening domestic health systems, including HIV response mechanisms. As Zimbabwe's economy continues to evolve, the country's capacity to sustain health programs, especially the HIV response, will increasingly rely on strengthening domestic financing mechanisms (2). With global health funding continuing to dwindle and the political dynamics in the global north growing more complex (3), enhancing domestic resource mobilization will be essential to gradually reduce dependence on external aid and improve resilience.

Zimbabwe's economy remains largely driven by its natural resources. Key mineral exports such as gold, platinum, lithium, chrome, iron, and nickel constitute a significant portion of the country's economic output (1). These minerals are in high demand globally, and their continued extraction, processing, and export have been integral to sustaining Zimbabwe's export earnings (4). Furthermore, the global surge in demand for lithium due to its critical role in battery production has positioned Zimbabwe as a key player in the global market (5). In addition to mineral exports, Zimbabwe has seen a steady increase in diaspora remittances, which have become a vital source of foreign currency (6, 7). In the nine months leading up to September 2024, Zimbabwe received approximately US$1.9 billion in remittances, reflecting a 16.5% increase compared to the same period in 2023 (8). Remittances have become a crucial financial lifeline for many households, driving the current account surplus and reducing poverty. Projections suggest that remittances will total US$2.49 billion by the end of 2024, with further growth to US$2.51 billion in 2025 (8). This steady inflow of funds supports household income and presents an opportunity for economic growth, including strengthening the health sector. Reflecting this potential, in 2024 the Zimbabwean government planned to allocate 11% of the total national budget to health expenditure, signaling a growing fiscal commitment to public health. Moreover, Zimbabwe's growing economic linkages with the Southern African Development Community (SADC) region offer opportunities for trade, investment, infrastructure development, resource pooling, and access to broader markets, all of which can accelerate its economic growth trajectory (9). This has been crucial for bolstering domestic revenues and enhancing Zimbabwe's ability to finance health services. As the economy continues to grow, Zimbabwe has the potential to leverage these inflows to augment further domestic funding for its health sector, including HIV response efforts.

Zimbabwe has made commendable progress in controlling its HIV epidemic. New infections have dropped substantially over the past decade, and the country has reached key health milestones in its HIV response (10, 11). Through effective prevention programs, widespread access to antiretroviral therapy (ART), and significant international support, Zimbabwe has moved from crisis management to a more stable and controlled epidemic. According to UNAIDS, Zimbabwe surpassed the 95-95-95 targets in 2023 (12). Despite these successes, Zimbabwe now faces a critical turning point in redefining HIV care, with a focus on sustainability beyond the UNAIDS 95-95-95 targets (13). HIV programs have evolved, and the country has reached a stage where HIV-specific programs, such as ART provision and PMTCT, often operating separately from the broader health system, need to be integrated. Moving forward, HIV prevention, treatment, and care should be fully incorporated into mainstream health services. This will create a more holistic approach to care and improve sustainability of HIV services and their accessible at all levels of the health system. Continuing with standalone HIV programs risks inefficiencies, overlap, and resource fragmentation, which could undermine efforts to provide comprehensive healthcare in the context of reducing donor funding for the HIV response.

To ensure integration and sustainability, Zimbabwe must increase its investment in the health system, including the HIV response. Key domestic revenue-generating initiatives, such as the AIDS levy and progressive taxation, are critical to achieving this. For example, a tax on sugary drinks generated US $38 million in the first 11 months of implementation, which was earmarked for the purchase of cancer diagnostic and treatment equipment for public hospitals (14). These levies have proven effective in raising substantial funds and establishing a domestic financial base for health programs. The Health Fund Levy of 5 cents for every dollar of airtime and mobile data, introduced in 2017, has enabled the Government of Zimbabwe to contribute US$ 7.6 million to the Health Development Fund for the purchase of essential medicines and health equipment (15). The AIDS levy, in particular, has played a crucial role in securing financing for the country's HIV response, especially as donor support has declined. Annually, the Zimbabwe HIV levy has raised approximately US$40 million to fund the National AIDS Council's HIV response (16).

Despite these efforts, there are still opportunities for improvement in how these funds are utilised. While the levies generate significant revenue, there are ongoing concerns regarding the efficiency and accountability of resource distribution. Some target populations, particularly vulnerable groups, may not fully benefit from these funds (17), often due to challenges such as inefficiencies in resource allocation, coordination issues among government agencies, and concerns about inadequate transparency and oversight. Just like other countries in the region Zimbabwe faces a decline in external donor funding and the HIV epidemic continues to evolve, it is increasingly important for the government to take full ownership of its HIV response. One potential avenue for generating additional domestic revenue is through a levy on diaspora remittances. Its anticipated that remittances will be approximately USD 5 billion annually in the near future, even a modest levy of 3%–5% could raise USD 150–250 million per year, which could be directed by the Zimbabwe government toward HIV programs and other essential health initiatives reducing the reliance on external donors. This approach would strengthen the domestic funding base and help ensure the long-term sustainability of Zimbabwe's HIV response.

While domestic revenue mobilization is critical to Zimbabwe's HIV response, the informal sector, private sector, and other stakeholders such as individuals, community organizations, and churches can also play significant roles in strengthening the country's health financing system. The informal sector, which comprises a large proportion of Zimbabwe's workforce, is largely untapped in terms of financial contributions to the health system. Strengthening policies that encourage the informal sector to participate in tax schemes, social health insurance, or voluntary levies could provide additional funding streams for the health system. Similarly, the private sector, through corporate social responsibility programs, can provide funding, resources, or services to support the HIV response. Churches and local community organizations, often with deep-rooted connections to vulnerable populations, can be crucial in implementing HIV awareness, prevention, and care initiatives. This multi-stakeholder engagement efforts results in a more inclusive, diverse financing base for its HIV programs.

One innovative approach could be the introduction of a targeted taxation framework on diaspora remittances, currently a major source of foreign exchange. Undoubtedly, a diaspora remittance levy would place additional financial strain on both remitters and recipients, and this raises important questions about fairness and feasibility. This is one example of how a major economic driver could potentially contribute to sustainable health financing. This approach may be considered as a starting point for dialogue on how to harness existing economic flows for public health purposes. Other sectors and revenue streams beyond remittances should also be explored for their potential to support the health system more broadly, not just the HIV response. Ultimately, any such strategy would need to be implemented in a way that is transparent, equitable, and informed by robust public engagement and feasibility assessments.

In parallel, Zimbabwe should explore the feasibility of local ARV drug manufacturing, not only to reduce dependence on imported medicines but also to position itself as a regional supplier to countries with limited domestic resource mobilization capacity. In 2020, Zimbabwe announced plans to begin local manufacturing of antiretroviral drugs through the state-owned national pharmaceutical, in collaboration with international partners. This initiative aimed to improve drug security, reduce costs, and support the national HIV response. However, no substantial progress has been reported since the announcement, highlighting the need for renewed commitment and investment in this area. Strengthening local pharmaceutical production capacity, beginning with essential medicines such as first-line ARVs, would represent a strategic step toward health system resilience. These strategies, if pursued responsibly and transparently, could offer a pathway toward a more self-reliant, resilient, and regionally supportive HIV response model.

To ensure the effective utilization of health financing, robust and enforceable accountability mechanisms must be put in place throughout the health system, particularly in the procurement and distribution of medicines and equipment, areas that have historically been vulnerable to mismanagement. This includes publishing detailed, accessible reports on health budgets and expenditures, conducting regular independent audits, and establishing citizen oversight committees to monitor fund allocation and service delivery. Strengthening whistleblower protections and enforcing legal consequences for corruption are also critical to deterring abuse. Targeted services for vulnerable groups, such as key populations affected by HIV, should be prioritized to ensure equitable benefit from available resources. Additionally, low-cost, community-based models of care, such as task-shifting, where community health workers assume roles traditionally performed by healthcare professionals, can help increase the accessibility and sustainability of HIV services (13). These models leverage local human resources and can help reduce costs, but only if they are implemented with transparency and ongoing evaluation. However, to prevent malpractices such as corruption and inefficiencies, there is a need for rigorous auditing and clear oversight mechanisms (18). Ultimately, trust in any innovative financing model will depend on the government's demonstrable commitment to accountability and the consistent, verifiable delivery of health services without leakage or diversion of funds.

Achieving Universal Health Coverage (UHC) is fundamental to the long-term success of Zimbabwe's HIV response. Health is a constitutional right in Zimbabwe, and this principle must guide the allocation of resources and the design of policies that address both HIV and broader health needs. The integration of HIV services into the broader health system aligns with the UHC objective by promoting a holistic and inclusive approach to healthcare.

As Zimbabwe continues to expand domestic financing for health, the government must prioritize policies and investments that guarantee equitable access to services for all, particularly marginalized and vulnerable communities. Additionally, the government must address political barriers that may hinder the expansion of the health financing landscape. One such barrier is the Private Voluntary Organisations Amendment Act, which imposes restrictive regulations on non-governmental organizations, reducing their ability to freely contribute to health programs. Some authors have argued that this bill may stifle civil society participation and discourage foreign and local actors from engaging in Zimbabwe's health sector (19, 20). To ensure the broadest possible participation in the HIV response, it is essential for the government to balance its regulatory oversight with an enabling environment for all stakeholders, including NGOs and private sector partners.

In conclusion, Zimbabwe's progress toward achieving middle-income status by 2030 is closely linked to its ability to finance its HIV response sustainably. While the country's economic outlook is promising, driven by mineral exports and diaspora remittances, the future of Zimbabwe's health system will depend on the government's capacity to mobilize domestic resources efficiently. Successfully integrating HIV services into the broader health system, along with more effective use of health leaves and exploring new revenue sources, will be crucial in ensuring the country stays on course in its fight against HIV. Achieving this will require Zimbabwe to prioritize greater accountability, transparency, and strategic investment in its health sector, ensuring that domestic and international resources are effectively deployed.
